# Psychosocial and organisational work factors as predictors of sickness absence among professionally active adults with common mental disorders

**DOI:** 10.1186/s12888-023-05020-3

**Published:** 2023-07-26

**Authors:** Magnus Helgesson, Klas Gustafsson, Constanze Leineweber

**Affiliations:** 1 Division of Insurance Medicine, Department of Clinical Neuroscience, Karolinska Institutet, Stockholm, SE-17177 Sweden; 2 Department of Public Health and Caring Sciences, Health Equity and Working Life, Uppsala University, Uppsala, SE-75237 Sweden; 3 Department of Psychology, Stockholm University, Stockholm, Sweden

**Keywords:** Sick leave, Common mental disorder (CMD), Psychosocial and organisational working conditions, Ill health

## Abstract

**Background:**

The incidence of sickness absence (SA) due to common mental disorders (CMDs) has increased in recent decades. It is hence important to elucidate how individuals with CMDs can maintain work. The aim was to analyse the relationship between psychosocial and organisational workplace factors and a spell of > 14 days of SA among persons with CMDs.

**Methods:**

Included were respondents of the Swedish Work Environment Survey (SWES) 1993–2013, diagnosed with a CMD up to five years before the interview in the SWES (n = 3,795). Relative Risk (RR) regression models with 95% Confidence Intervals (CIs) analysed associations between psychosocial-, and organisational workplace factors and a subsequent spell of SA > 14 days.

**Results:**

Low control over work (RR:1.16; CI:1.01–1.35), job strain (RR:1.25; CI:1.04–1.49), no flexible working hours (RR:1.25; CI:1.08–1.45) or no possibility to work from home (RR:1.37; CI:1.13–1.66) were significantly related to an increased risk of SA. Persons diagnosed with depression experiencing job strain had the highest increased risk of SA (RR:1.55; CI: 1.07–2.25).

**Conclusions:**

A sustainable work-life among working individuals with CMDs can be provided by reducing job strain, and if possible, by increasing flexibility regarding workplace and working hours. This may prevent spells of SA, and hereby increase productivity.

**Supplementary Information:**

The online version contains supplementary material available at 10.1186/s12888-023-05020-3.

## Background

Nearly 30 per cent of the world’s population meets the criteria for having a common mental disorder (CMD), that is depression, anxiety- or stress-related disorders, at least once during their working life [1]. Since CMDs often affect workability, these disorders are one of the most common causes of sickness absence and disability pension in Europe [2–4]. The incidence of CMDs and sickness absence due to CMDs have gradually increased during the 2000s, especially the occurrence of stress-related illnesses has increased significantly since 2010 [5]. One study shows that half of the patients with a first episode of a CMDs in young adulthood have a poor connection to the labour market [6]. Also, the inability to work because of a CMD is very costly to both employers and society, not least for the individuals themselves who are risking permanent welfare dependence [7]. Therefore, it is important to determine factors that hinder or facilitate work participation in this large group suffering from CMDs.

Sickness absence due to CMDs, especially stress-related disorders, often has a link to poor working conditions [8–13]. On the one hand, factors such as high control, low demands and good social support form a ‘good’ psychosocial work environment can provide a healthy sustainable work-life [14]. On the other hand, a negative psychosocial work environment, for example, characterized by a combination of high demands and a low degree of control and social support, can lead to periods of sickness absence and ultimately early retirement [12, 15–18]. Also, organisational workplace factors such as inconvenient working hours, that is working in evenings, nights, and weekends, flexible working hours or the possibility of working from home can affect the person’s ability to work. In addition, sex, age and educational level can affect sickness absence among persons with CMDs. Since the mid-1990s, two-thirds of those with sickness absence benefits are women [19] and it has been shown that those with a low educational level have a higher propensity of being absent from work due to illness [6]. Therefore, these factors must be considered when performing studies on work factors and sickness absence.

To conclude, the relationship between CMDs and work-related factors is complex and the scientific knowledge regarding these factors is limited [20]. At the same time, sickness absence due to CMDs is increasing in many European countries [4]. The identification of the work-related factors that are associated with a high sickness absence rate among people with CMDs is therefore highly warranted.

The aim was to study the relationship between psychosocial-, and organisational workplace factors and sickness absence three years after participation in the Swedish Work Environment Survey (SWES), and if there are variations regarding these relationships in different CMD diagnoses.

## Materials and methods

Several indicators of working conditions were obtained from the SWES and the Labour Force Surveys (LFS) from 1993 to 2013. The SWES covers a broad range of work conditions and has been conducted every second year since 1989 [21]. In this study, we have used data regarding psychosocial work exposures from the SWES and data regarding organisational work exposures from the LFS for the years 1993 to 2013 [22]. The items in the SWES and LFS have been validated by re-interview methodology [23]. Good reliability has been obtained and results were reported in previous studies [24]. The response rate of the SWES spans from 82% in 1993 to 66% in 2013 [22]. In total, SWES consist of a representative sample of the Swedish employed population aged 16 to 64. For the years 1993 to 2013, there were 100,719 participants.

### Study population

The study population was drawn from a subsample of those participating in any of the SWES 1993–2013 [25] and who had been diagnosed with a CMD up to five years before they answered the SWES. Data regarding CMDs were obtained from the National Patient Register [26] and were defined as a diagnosis for depression, anxiety- or stress-related disorders taken from inpatient or outpatient health care in Sweden. According to the International Statistical Classification of Diseases (ICD), they correspond to the codes in version 10 (ICD-10): F32 – F43; and version 9 (ICD 9): 296, 298, 300, 301, 308, 309 or 311 (n = 1237). The largest share of participants was diagnosed with a stress-related diagnosis (ICD-10: F43, ICD-9: 308 and 309, n = 556 (14.6%)) followed by anxiety disorder (ICD-10: F40-F42, ICD-9: 300, n = 370 (9.8%), and depression (ICD-10: F33-F39, ICD9: 296, 298, 301 and 311, n = 230 (6.1%)). Following the example of previous studies, also those who were prescribed antidepressants (ATC-code N06A) up to five years before the interview in the SWES were added [6]. This group made out the largest group of the sample, n = 2,639 (69.5%). The inclusion process was hierarchical where individuals with a record of a diagnosis of CMD were included first, and in a second step, those without a diagnosis but with a record of prescription of antidepressant medication were included. After the exclusion of those who received disability pension before participating in SWES, 3,795 persons remained in the study population.

### Outcome measure - sickness absence

In Sweden, all individuals from 16 years and onwards, with an income above a certain level, can receive benefits when having sickness absence. The employer is responsible for the payment of benefits during the first 14 days and this period is not covered by registers. Moreover, there is one qualifying day (3 or 30 days depending on chosen fee among self-employed) without benefits. Thus, sickness absence in this study covers sick-leave periods of 15 days or more. Moreover, two days of half-time sickness absence were counted as one net day of sickness absence. Information on sickness absence up to three years after participation in SWES was derived from the “Longitudinal integrated database for health insurance and labour market studies” (LISA) and linked to survey data by the unique national identification number.

### Psychosocial work exposures

Job strain was defined following the work demand-control model [14, 27] with several variables serving as proxies. Four variables were used as indicators of job demands and job control, respectively [28]. To follow the questions stated below, the SWES from 2015 is attached as a supplementary file (SWES 2015). Indicators of high job demands, with cut-offs given in parenthesis after the respective item, were a) “have to miss lunch, work late, or take work home’’ (≥ 1 day of 2), b) ‘‘do not have time to talk or even think about something other than work’’ (≥ ¾ of the time), c) ‘‘the work requires your full attention and concentration’’ (nearly all the time) or d) ‘‘have far too much to do at work’’ (too much to do, response 1). Indicators of low job control were e) “are you able to determine when various working duties are to be carried out’’ (no, not at all), f) ‘‘participate in decisions on the arrangement of your work’’ (≤ mostly not), g) ‘‘have the opportunity to determine your work pace’’ (≤ 1/10 of the time), or h) ‘‘have too little influence at work’’ (≤ too little influence, response 1 and 2). Item a) was answered on a five-point scale reaching from 1 = every day to 5 = not at all/rarely in the last three months. Items b), c), and g) were answered on a six-point scale reaching from 1 = Nearly all of the time to 6 = No, not at all. Item d) was answered on a five-point scale reaching from 1 = far too much to do to 5 = far too little to do. In similarity, item h) was answered from 1 = too little influence to 5 = too much influence. Items e) and f) were answered on a four-point scale reaching from 1 = always to 4 = no, not at all. The upper quartile was defined as having been exposed to high job demands and low job control, respectively. If a person answered positively to at least two of the indicators of work demands or control, this indicated high job demands or low job control, respectively [28].

Social support was indicated by two items “Can you receive support and encouragement from your superiors when your work becomes troublesome?” and “Can you receive support and encouragement from your fellow workers when your work becomes troublesome?”, answered on a 4-point Likert scale from 1 = always to 4 = never. Receiving social support (always or most of the time) from both colleagues and superiors was calculated as receiving social support, while all other options were coded as not receiving social support.

Further, one item measured the atmosphere at work (open or closed) “Are you reluctant to express critical views in the workplace regarding your working conditions?” answered on the same scale as described for items e and f (see above). Having this possibility for always or most of the time indicated an open atmosphere. Two other indicators of psychosocial work were: “Do you feel ill at ease and downhearted as a result of the difficulties you face at work?” and “At the end of your workday, do you feel that your work input is inadequate?” answered on the same scale as item a (see above). Feeling badly or experiencing despair at least one day of the week (1 day of 5) indicated a poor work environment.

### Organisational workplace factors

The indicators of the organisational work environment factors were work flexibility, usually working at home, inconvenient working hours, and full-time or part-time work. Flexibility at work was indicated by two items. “In general, are you able to decide your working hours, within certain limits?” with response options “Yes, I have flextime (begin and end within certain fixed times, but not at an exact time point)” and “Yes, I have relatively free working hours in another way” indicating flexibility, while “No, in general, I cannot change my working hours” was coded as not having flexibility.

Another organisational workplace factor was “How much of your normal working time do you usually work at home”. Having this possibility at least some hours a week was indicating the possibility to work from home.

Inconvenient working hours were indicated by the need to work evenings, nights, or weekends (Saturday or Sunday). If any of this was the case, it was coded as inconvenient working hours.

### Confounders

Age, sex, educational level, and year of participation in SWES were considered potential confounding variables. Age, sex, and educational level were obtained from the LISA database [29]. Age was coded into five age groups (16 to 25 years, 26 to 35 years, 36 to 45 years, 46 to 55 years, and 56–64 years). Sex is binary, men and women. Educational level was based on the Swedish classification system of education (SUN2000) and coded into three groups (low (elementary school), medium (upper secondary school), and high (university)).

### Statistical methods

Relative risk (RR) regression models with a 95% confidence interval (CI) were calculated to analyse associations between psychosocial and organisational workplace factors and subsequent sickness absence three years after the interview in SWES using *proc genmod* in SAS (SAS Institute Inc. 2013. SAS® 9.4.) [30]. In the first step, crude models were calculated. In the next step, we controlled for the year of interview. In a third model, we additionally controlled for age, sex, and educational level. Further, relative risk regression models stratified by the three diagnoses (stress, anxiety, and depression) were used.

## Results

### Characteristics

Most of the participants in the study were aged 46 years or above (Table 1). More than 2 out of 3 were women and very few study participants had a low educational level. Around 17% had at least one spell of sickness absence exceeding 14 days during the follow-up period of three years after participation in SWES.

**Table 1:** Description of the study group (n = 3795 persons with common mental disorders (CMDs))

	Total	No sickness absence in follow-up	Sickness absence in follow-up
	**n (%)**	**n (%)**	**n (%)**
**Total**	**3795 (100)**	**3145 (82.9)**	**650 (17.1)**
Age			
16–25	172 (4.5)	150 (4.8)	22 (3.4)
26–35	568 (15.0)	462 (14.7)	106 (16.3)
36–45	957 (25.2)	811 (25.8)	146 (22.5)
46–55	1190 (31.4)	966 (30.7)	224 (34.5)
56–64	908 (23.9)	756 (24.0)	152 (23.4)
Sex			
Women	2596 (68.4)	2094 (66.6)	502 (77.2)
Men	1199 (31.6)	1051 (33.4)	148 (22.8)
Educational level			
Low (Elementary school)	556 (15.3)	461 (15.3)	95 (15.2)
Medium (Upper secondary school)	1783 (49.1)	1469 (48.8)	314 (50.3)
High (University)	1294 (35.6)	1079 (35.9)	215 (34.5)
Source of diagnosis for CMD			
Hospital diagnosis	647 (17.1)	524 (16.7)	123 (18.9)
Prescribed medicine	2558 (67.4)	2148 (68.3)	410 (63.1)
Both	590 (15.6)	473 (15.0)	117 (18.0)

### Psychosocial workplace factors

Experiencing low control over work (RR = 1.16 95% CI: 1.01–1.35) and experiencing high job strain (RR = 1.25 95% CI: 1.04–1.49) were related to an increased risk of long-term sickness absence (Table 2). Adjustments for age, sex and educational level just altered the results slightly. Among persons with depression, the risk of sickness absence was especially high when experiencing high job strain 1.55 (1.07–2.25) (Table 3).

**Table 2:** Relative risk (RR) for a spell of sickness absence > 14 days with a 95% confidence interval (CI), for individuals diagnosed with common mental disorders (CMD) for various work environment factors (n = 3795)

Exposure variables		Number/cases	^a^ Model 1	^b^ Model 2	^c^Model 3
			RR (95% CI)	RR (95% CI)	RR (95% CI)
**Psychosocial factors**					
*Work demands*	Low	2609/425	1	1	1
	High	1185/225	**1.16 (1.01–1.35)** ^*^	**1.17 (1.01–1.36)** ^*^	1.13 (0.98–1.31)
*Work control*	High	2586/413	1	1	1
	Low	1208/237	**1.23 (1.06–1.42)** ^*^	**1.22 (1.06–1.42)** ^*^	**1.16 (1.01–1.35)** ^*^
*Job strain*	No	3241/531	1	1	1
	Yes	553/119	**1.31 (1.10–1.57)** ^*^	**1.32 (1.11–1.58)** ^*^	**1.25 (1.04–1.49)** ^*^
*Work support*	High	1606/278	1	1	1
	Low	2189/372	1.02 (0.88–1.17)	1.01 (0.88–1.11)	1.02 (0.88–1.18)
*Feel ill at ease*	No	2871/485	1	1	1
	Yes	840/149	1.05 (0.88–1.24)	1.06 (0.90–1.25)	1.05 (0.88–1.24)
*Feel your work is inadequate*	No	2498/396	1	1	1
	Yes	994/178	1.13 (0.96–1.33)	1.13 (0.96–1.33)	1.11 (0.94–1.31)
*An open atmosphere at work*	Yes	2700/431	1	1	1
	No	884/152	1.15 (0.98–1.35)	1.14 (0.97–1.35)	1.14 (0.97–1.35)
**Organisational factors**					
*Flexible working hours*	Yes	2179/332	1	1	1
	No	1520/297	**1.28 (1.11–1.48)** ^*^	**1.28 (1.11–1.47)** ^*^	**1.25 (1.08–1.45)** ^*^
*Working from home*	Yes	2814/515	1	1	1
	No	897/115	**1.43 (1.18–1.72)** ^*^	**1.41 (1.17–1.70)** ^*^	**1.37 (1.13–1.66)** ^*^
*Inconvenient working hours*	No	1956/315	1	1	1
	Yes	954/173	1.12 (0.95–1.33)	1.13 (0.95–1.33)	1.10 (0.93–1.31)
*Fulltime work*	No	1356/239	1	1	1
	Yes	2325/392	0.96 (0.83–1.11)	0.96 (0.83–1.11)	1.09 (0.93–1.28)

^a^ Model 1: Crude. ^b^ Model 2: Model 1 + year of interview, ^c^ Model 3: Model 2 + age, sex, educational level

*Significant figures (p < 0.05)

**Table 3:** Relative risk (RR) for a spell of sickness absence > 14 days with a 95% confidence interval (CI) for individuals (n = 3,795) with common mental disorders (CMDs) stratified by three diagnoses. Results for the crude model are shown

		Depression (n = 556)			Anxiety (n = 360)			Stress (n = 230)		
Exposure variables		Number/ cases	Crude	Adjusted*	Number/ cases	Crude	Adjusted*	Number/ cases	Crude	Adjusted*
**Psychosocial factors**			**RR**	**RR (95% CI)**		**RR**	**RR (95% CI)**		**RR**	**RR (95% CI)**
*Work demands*	Low	380/75	1	1	272/48	1	1	138/19	1	1
	High	176/42	1.21	1.16 (0.83–1.63)	98/20	1.16	1.15 (0.73–1.83)	92/15	1.18	1.08 (0.58–2.03)
*Work control*	High	371/73	1	1	241/49	1	1	148/20	1	1
	Low	185/44	1.21	1.23 (0.87–1.70)	129/19	0.77	0.80 (0.49–1.29)	82/14	1.26	1.19 (0.63–2.22)
*Job strain*	No	476/92	1	1	322/60	1	1	184/25	1	1
	Yes	80/25	**1.62** ^**^	**1.55 (1.07–2.25)** ^**^	48/8	0.89	0.90 (0.31–1.76)	46/9	1.44	1.37 (0.68–2.74)
*Work support*	High	313/66	1	1	212/40	1	1	114/18	1	1
	Low	243/51	1.00	0.95 (0.69–1.32)	158/28	0.94	0.96 (0.62–1.48)	116/16	0.87	0.94 (0.51–1.74)
*Feel ill at ease*	No	374/78		1	278/56		1	162/20	1	1
	Yes	140/31	1.06	1.07 (0.74–1.55)	83/10	0.60	0.61 (0.33–1.15)	57/12	1.71	N/A***
*Feel your work is inadequate*	No	293/59	1	1	229/40	11	1	229/40	1	1
	Yes	131/26	0.98	0.96 (0.63–1.46)	94/12	0.73	0.80 (0.44–1.48	137/20	1.11	0.96 (0.51–1.83)
*An open atmosphere at work*	Yes	348/74	1	1	247/43	1	1	155/22		1
	No	131/25	0.90	0.86 (0.57–1.31)	90/14	0.89	1.03 (0.59–1.82)	70/10	1.01	1.05 (0.53–2.08)
**Organisational factors**										
*Flexible working hours*	Yes	318/63	1	1	191/32	1	1	131/15	1	1
	No	212/46	1.10	1.10 (0.78–1.56)	165/32	1.16	1.23 (0.79–1.90)	94/19	1.76	1.67 (0.91–3.05)
*Working from home*	Yes	127/21	1	1	93/14	1	1	94/5	1	1
	No	405/88	1.31	1.31 (0.84–2.02)	265/50	1.25	1.34 (0.78–2.29)	171/29	1.90	N/A***
*Inconvenient working hours*	No	208/43	1	1	166/31	1	1	93/16	1	1
	Yes	115/24	1.01	1.06 (0.67–1.66)	96/11	0.61	0.64 (0.33–1.22)	53/12	1.32	1.06 (0.55–2.02)
*Fulltime work*	No	222/40	1	1	114/21	1	1	69/12	1	1
	Yes	313/73	1.29	1.42 (0.99–2.04)	244/44	0.98	0.98 (0.60–1.60)	148/20	0.78	0.97 (0.50–1.87)

*Controlled for age, sex, educational level

**Significant figures (p < 0.05)

^***^N/A: The specified model did not converge due to few numbers

### Organisational workplace factors

Having no flexibility regarding working hours (RR: 1.25 95% CI: 1.08–1.45) and having no possibility to working from home (RR: 1.37 95% CI: 1.13–1.66) was connected to having a higher risk of experiencing a spell of over 14 days of sickness absence in the follow-up period (Table 2). Also here, adjustments for age, sex and educational level had just a slight effect.

There was an indication that individuals with stress-related disorders had an especially high risk of a spell of sickness absence exceeding 14 days when exposed to no flexibility regarding working hours (RR: 1.67 95% CI: 0.91–3.05) (Table 3). Due to the low sample size regarding the sub-analyses on specific diagnoses, the confidence intervals are wide, and these results are therefore more uncertain and must be interpreted with caution.

## Discussion

### Main findings

Psychosocial factors such as low control over work and having high job strain were significantly related to an increased risk of having a period over 14 days of sickness absence three years after the exposure among workers diagnosed with CMD. Job strain seemed most detrimental to individuals with depression. Further, the risk of organisational workplace factors such as no flexibility regarding working time or no possibility to work from home were related to an increased risk of having a period over 14 days of sickness absence.

The relative risk of having a spell of sickness absence that exceeded 14 days was increased for individuals exposed to several psychosocial or organisational work exposures. The increases in the risk of sickness absence due to job strain in this study (RR: 1.25; CI: 1.04–1.49) were, however, rather modest compared to meta-analyses on other populations, both compared to individuals in general (RR: 1.44; CI: 1.29–1.60) as well as on individuals with mental illness (RR: 1.61; CI: 1.19–2.17) [31]. Reasons for this discrepancy might primarily be that we have a selected working population that was diagnosed or had treatment for CMDs during the baseline year although individuals with baseline sickness absence were excluded from the study. Individuals with CMDs have in general been reported to have an increased risk of work disability [4, 6].

### Psychosocial workplace factors

Foremost experiencing high job strain, i.e., high job demands and low job control combined, were associated to having spells of sickness absence exceeding 14 days. Also, the specific items of job strain, that is low job control and high job demands (although not statistically significant in the final model) were connected to having at least one period over 14 days of sickness absence during the follow-up of three years. Several other studies have reported that low control and high demands combined are a real risk for subsequent work disability [31]. A systematic review also concludes an association between job strain and depression [17]. We can in this study conclude that individuals with depression who were exposed to high job strain had an especially high risk of having a spell exceeding 14 days of sickness absence three years after the exposure. It is well known that high job strain at work is detrimental to workability [14, 31] and a recent review has stated that health interventions alone may not be sufficient to avoid work disability among individuals with CMDs [13]. In addition, work-related interventions targeting detrimental work exposures are needed for a successful return to work in this group [13]. Providing a good work environment can therefore prevent long spells of sickness absence, which gives an economic incitement to increase productivity by improving the work environment.

The supporting role of colleagues and supervisors has in studies seen to be protective against future sickness and a poor team climate may increase the risk of sickness absence in a workgroup [32, 33]. In this study, there was no significant association between social support at work and having at least one period over 14 days of sickness absence during the follow-up. This might be explained by the fact that we in this study only examined longer periods of sickness absence. Having the ability to take shorter periods of sickness absence may be protective for longer spells of sickness absence [34]. In a good work climate, the employees are recommended to be on short-term sickness absence when sick. Another explanation might be that support mostly might be emotional, not instrumental [35]. This means that work tasks not will be taken over by colleagues when an employee is absent. If the work is piled up while on sickness absence, the risk of future long-term sickness absence probably will increase by the increased workload. Therefore, an indication of having social support at work may not always be protective against later spells of sickness absence.

### Organisational workplace factors

Organisational work exposures such as no flexible working hours and no possibility to work from home seem to increase the risk of sickness absence among individuals with CMDs. Those who could not work from home had a higher risk of having a spell of sickness absence exceeding 14 days in the follow-up compared to those who could work from home. A Finnish study has reported that control over daily working hours and days off could moderate the association between job strain and sickness among public sector employees [36]. Also, when including working individuals with CMDs, it seems to be important to have the possibility to have flexible working hours. Such flexibility would allow individuals with CMDs to adapt their work demands to their daily workability.

Also, not having the possibility to work from home was connected to a higher risk of having a spell of sickness absence over 14 days. Time for commuting to work and being regularly at the workplace may for some individuals be a burden that may harm recovery. However, there is, in the literature evidence for both positive and negative consequences on well-being by the possibility to work from home [37]. Individual needs are therefore important for choosing who can work from home or not [37]. The lack of the possibility to work from home can also be a proxy for a poor work environment, where the employer and the employee do not have good communication [37]. There might also be large differences in the possibility to work from home between different workplaces and occupations. It is unlikely that blue-collar workers, e.g., bus drivers can work from home, but more likely that white-collar workers, e.g., a clerk, can work from home. Studies have shown that white-collar workers in general have a lower risk of sickness absence, and the higher ability to work from home when feeling ill can explain part of the difference in sickness absence [36]. We could also see that especially among individuals with stress-related disorders there was an even higher tendency that working from home could prevent periods of sickness absence. This might help target specific work interventions to persons diagnosed with CMDs. Here, the symptomatic picture of stress-related disorders might be helped by having flexibility regarding both working time and working place. Studies report that losing social interaction by working from home may be a risk factor for depression [38], regarding stress-related disorders the situation is less clear. This is therefore a complex matter that requires individual strategies to find a good balance between working from home and the workplace.

### Strengths and limitations

The strengths of the present study were the use of a representative sample of the Swedish employed population, high-quality data from Swedish registers and a prospective design.

Limitations were that we in this study had a small sample to do analyses on periods exceeding > 14 days of sickness absence, e.g. spells over 60 or 90 days which in many studies are regarded as long-term sickness absence. There might hence be differences when measuring longer periods of sickness absence as the protective effect of a good work environment might differ regarding longer and shorter periods of sickness absence. All of the study population had employment at the time of the interview in the SWES. This means that the participants, due to the healthy workers’ effect, probably are healthier than the general population with CMDs [39]. The external validity may therefore be limited only to individuals with CMDs having a strong attachment to the labour market.

## Conclusions

A sustainable work-life among working individuals with CMDs can be provided by reducing job strain, and if possible, also by increasing flexibility regarding workplace and working hours. This can prevent long spells of sickness absence, and hereby increase productivity and avert costs among employers.

## Electronic supplementary material

Below is the link to the electronic supplementary material.

Supplementary Material 1: SWES 2015

## Data Availability

These data cannot be made publicly available due to privacy regulations. According to the General Data Protection Regulation, the Swedish law SFS 2018:218, the Swedish Data Protection Act, the Swedish Ethical Review Act, and the Public Access to Information and Secrecy Act, these types of sensitive data can only be made available for specific purposes, including research, that meets the criteria for access to this type of sensitive and confidential data as determined by a legal review. Readers may contact Assistant Professor Magnus Helgesson (magnus.helgesson@ki.se) regarding the data.
